# Preclinical Dementia Risk Estimation: Attitudes and Views of Relatives of Dementia Patients – The PreTAD‐Study

**DOI:** 10.1002/alz.087313

**Published:** 2025-01-09

**Authors:** Michelle Gerards, Annika Baumeister, Constanze Hübner, Federica Ribaldi, Yahveth Cantero‐Fortiz, Julia Braun, Mercè Boada, Giovanni B. Frisoni, Björn Schmitz‐Luhn, Carolin Schwegler, Christiane Woopen, Frank Jessen, Ayda Rostamzadeh

**Affiliations:** ^1^ Department of Psychiatry and Psychotherapy, Medical Faculty, University of Cologne, Cologne Germany; ^2^ Center for Life Ethics, University of Bonn, Bonn Germany; ^3^ Geneva Memory Center, Department of Rehabilitation and Geriatrics, Geneva University Hospitals, Geneva Switzerland; ^4^ Laboratory of Neuroimaging of Aging (LANVIE), University of Geneva, Geneva Switzerland; ^5^ Ace Alzheimer Center Barcelona – International University of Catalunya (UIC), Barcelona Spain; ^6^ Ace Alzheimer Center, Barcelona Spain; ^7^ Network Center for Biomedical Research in Neurodegenerative Diseases (CIBERNED), Madrid Spain; ^8^ University of Geneva, Geneva Switzerland; ^9^ Faculty of Arts and Humanities, University of Cologne, Cologne Germany; ^10^ German Center for Neurodegenerative Diseases (DZNE), Bonn Germany; ^11^ Excellence Cluster on Cellular Stress Responses in Aging‐Associated Diseases (CECAD), University of Cologne, Cologne Germany

## Abstract

**Background:**

Due to further development of diagnostic methods of early‐stage diagnosis of Alzheimer’s disease (AD) and new disease‐modifying treatment options that require early diagnosis, a new focus on predictive and preventive medicine arises. With progress in AD dementia risk estimation, guidelines for counseling, considering individual aspects of those affected, are becoming more important. As part of the trinational project PreTAD (The Predictive Turn in Alzheimer’s Disease: Ethical, Clinical, Linguistic and Legal Aspects) anticipated effects of AD dementia risk estimation for first‐degree relatives of people with AD dementia are evaluated.

**Method:**

In the PreTAD project, the topics risk understanding, and anticipated effects of risk estimation were surveyed. First‐degree relatives of people with AD dementia were confronted with one of the following hypothetical AD dementia risk scenarios: 1) higher risk due to abnormal biomarker results or 2) a lifetime risk comparable to the general population. First results of the main study with 50 participants in Germany are reported.

**Result:**

Almost all participants would undergo AD dementia risk estimation if offered. The preferred method was blood sampling compared to other diagnostic methods. Anticipated effects of AD dementia risk estimation were disadvantages at work, on one’s financial situation and when taking out insurance policies. Fewer disadvantages were expected on relationships with close others. Participants confronted with a higher risk of developing AD dementia were more likely to feel diagnosed with a disease and were less likely to share test results with close others. Those confronted with a higher risk were more likely to be willing to change their lifestyle, and make changes in further life planning. Anticipated effects on mental health (e.g. depressed mood, increased anxiety) were higher when confronted with a higher risk of AD dementia.

**Conclusion:**

First results show that dementia risk estimation is desired by first‐degree relatives of people with AD dementia and may have an effect on various elements of personal life, including lifestyle, further life planning and mental health. Findings of the PreTAD project will help to inform the process of counseling and clinical management within preclinical predictive AD diagnosis.

